# Progress in Methylxanthine Biosynthesis: Insights into Pathways and Engineering Strategies

**DOI:** 10.3390/ijms26041510

**Published:** 2025-02-11

**Authors:** Tongtong Jiang, Shangci Zuo, Chang Liu, Wanbin Xing, Pengchao Wang

**Affiliations:** 1 School of Life Science, Northeast Forestry University, Harbin 150040, China; 19806011960@nefu.edu.cn (T.J.); 16645136221@nefu.edu.cn (S.Z.); lc1677315800@nefu.edu.cn (C.L.); 2 Key Laboratory for Enzyme and Enzyme-Like Material Engineering of Heilongjiang, College of Life Science, Northeast Forestry University, Harbin 150040, China; 3 Aulin College, Northeast Forestry University, Harbin 150040, China; wanbinxing@nefu.edu.cn

**Keywords:** biotechnology, biosynthesis, methylxanthines, N-demethylation, enzyme engineering, biocatalysis

## Abstract

Methylxanthines are ubiquitous purine alkaloids in nature and have rich biological activities and functions. Today, the demand for methylxanthine is increasing but its production is low. This issue prevents its widespread use in many industrial fields, such as pharmaceuticals, food manufacturing, and chemical engineering. To address these issues, this review provides a comprehensive and systematic exploration of methylxanthines, delving into their biological structures, detailed biosynthetic pathways, and the latest research trends. These findings serve as valuable references for researchers, fostering advancements in the optimization of synthesis processes for methylxanthines and their derivatives and promoting their application across diverse industrial fields, such as medicine, food, and chemical engineering. By bridging fundamental research and practical applications, this work aims to advance the understanding of methylxanthine compounds, enhance their production efficiency, and contribute to healthcare and technological progress.

## 1. Introduction

**Figure 1:**
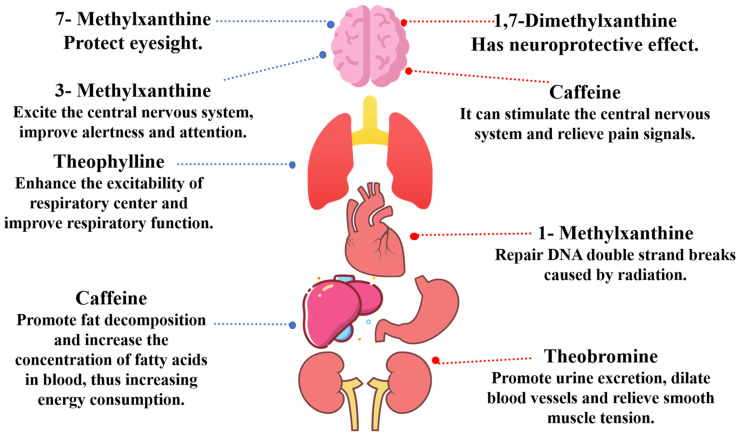
Functions of methylxanthine derivatives. Methylxanthine derivatives, including 7-methylxanthine, 3-methylxanthine, 1-methylxanthine, 1,7-dimethylxanthine, theobromine, theophylline, and caffeine, exhibit diverse bioactivities within various human physiological systems.

Methylxanthines are a kind of purine alkaloid with unique chemical structures that perform many physiological functions [1]. The molecular structure of different types of methylxanthines is characterized by containing specific substituents on purine rings, which results in a wide range of biological activities in vivo [2]. These compounds can participate in various physiological processes, such as nerve conduction, muscle contraction, and cardiovascular function regulation, and are crucial for maintaining the normal physiological functions of organisms (Figure 1) [3,4,5,6].

Methylxanthines are widely present in nature, especially in foods such as tea, coffee, and cocoa, where they are abundant [7,8,9,10]. Based on their chemical structures and the varying positions of substituents, methylxanthine can be categorized into three derivatives: monomethylxanthine, dimethylxanthine, and trimethylxanthine. These compounds not only are widely distributed in nature but can also be prepared by artificially synthesized methods to meet the needs of different industries and applications [11,12,13,14,15].

From the biological point of view, methylxanthine is involved in many biological processes. In the field of medicine, the study of methylxanthine and its derivatives provides a potential target for the development of new drugs [16,17,18,19,20,21]. In addition, it also has important applications in food and chemical industries. Therefore, it is very important to study the characteristics of methylxanthine and its influence on the human body for scientific and technological progress in related fields [21,22,23]. In recent years, with the rapid development of metabolic engineering technology, significant progress has been made in the study of methylxanthine biosynthesis. By analyzing the methylxanthine biosynthesis pathway, researchers have revealed the process for the synthesis of these alkaloids in plants, providing a theoretical basis for improving yield and optimizing quality through genetic engineering and metabolic engineering [24,25,26,27,28,29]. These research results not only help further elucidate the methylxanthine biosynthetic mechanism but also provide key support for the development of new drugs and the expansion of their application [30,31].

In this review, the latest progress in research on methylxanthine biosynthesis is reviewed; the contents are carefully organized into four parts for in-depth discussion. First, we introduce the chemical structure and physiological function of methylxanthines in detail, as well as their distribution and classification in nature. Second, we summarize the different synthetic pathways for methylxanthines, including natural synthesis and chemical synthesis, and analyze the factors affecting methylxanthine yield and quality [32,33,34]. Next, we focus on the latest progress in research on the biosynthesis of methylxanthines, exploring the mechanism of biosynthesis, the functions of key enzymes, and the application of metabolic engineering strategies to improve yield and optimize quality. Finally, we highlight the current challenges and future research directions, providing references and lessons for further research and application of methylxanthines.

## 2. Biological Structures and Bioactivities of Methylxanthine Derivatives

Xanthine is an important purine base, and its structure features a pyrimidine ring and an imidazole ring, which are skillfully connected by a carbon-nitrogen bond. Xanthine is a key molecule in organisms; it is often used as a precursor for the synthesis of other purine compounds in the purine metabolism network, plays the role of a transport hub, and is transformed into many purine compounds with unique physiological functions through enzymatic reactions [35,36,37,38]. The purine compounds produced from xanthine are diverse and have different functions. These derivatives include adenine and guanine, which are the key components of DNA and RNA and are very important for stable transmission and accurate expression of genetic information, ensuring the continuity and stability of biological genetic information [38,39,40,41]. Methylxanthines are also important xanthine-derived products.

Methylxanthines are xanthine derivatives obtained by methylation [13,42]. The nomenclature of methylxanthines is based on the positions of methyl substitutions on the purine ring. These methyl groups not only alter the chemical properties of the molecules but also significantly influence their physiological activities and metabolic pathways (Figure 1). Variations in the positions and numbers of methyl groups among members of the methylxanthine family result in diverse biological activities and metabolic behaviors [43]. In monomethyl xanthine, 3-methylxanthine (3-MX) is a prominent example. It is characterized in that the N-3 position of the purine ring is connected with methyl, which makes it a methylated derivative of xanthine [44]. Functionally, 3-MX has various pharmacological effects [45]. It can effectively stimulate the central nervous system and improve an individual’s alertness and attention level. This effect may be attributed mainly to its inhibitory effect on adenosine receptors. This mechanism helps reduce the inhibitory effect of adenosine on neurons, thus enhancing the transmission of nerve signals and brain activity [46]. Additionally, 3-MX can influence lipid metabolism and energy expenditure, with moderate consumption potentially aiding in weight management [47,48].

Another notable monomethylxanthine is 1-methylxanthine (1-MX), a caffeine derivative and a urinary metabolite of caffeine and theobromine in humans [49]. In the molecular structure of 1-MX, there is a purine ring with a methyl group at the N-1 position. This unique molecular configuration endows 1-MX with unique chemical characteristics and pharmacological activities. Research has shown that 1-MX markedly enhances the sensitivity of RKO (a human colorectal cancer cell line) human colorectal cancer cells harboring the wild-type p53 (tumor protein 53) gene to radiotherapy. Specifically, 1-MX plays its role by directly inhibiting the repair process of DNA double-strand breaks (DSB) [50]. Under normal circumstances, when cells are subjected to external damage factors such as radiotherapy, DNA double-strand breaks occur, which is an important form of cell damage. In order to maintain the stability of the genome, cells will start a series of complex repair mechanisms to try to repair these injuries. However, 1-MX can interfere with this process, making the damaged DNA unable to be repaired in time and effectively, thus increasing the risk of cancer cell death.

7-Methylxanthine (7-MX) is another monomethylxanthine, characterized by the attachment of a methyl group at the N-7 position of the xanthine ring. This compound is a metabolic product of caffeine and theobromine in humans and is present in purine form in urinary calculi. It has significant physiological and pharmacological importance [51]. In recent years, 7-MX has garnered increasing attention. Scientific studies have demonstrated its marked therapeutic effects on deprivation-induced myopia in animals such as pigmented rabbits, guinea pigs, and rhesus monkeys [52,53,54]. Moreover, several clinical trials have confirmed that 7-MX effectively inhibits and stabilizes the progression of myopia in adolescents, offering a new approach to treating patients with this condition [55,56]. Additionally, comprehensive toxicological evaluations have verified the safety of 7-MX, highlighting its lack of genotoxicity, mutagenicity, and cytotoxicity [57,58]. These findings establish 7-MX as a safe and reliable long-term oral agent for mitigating myopia progression [59].

Dimethylxanthines include theobromine, theophylline, and paraxanthine (PX). Theobromine is characterized by methylation at the N-3 and N-7 positions of the xanthine molecule. Theobromine is abundant in nature, especially in cocoa beans. In chocolate, there are 40 to 500 mg of theobromine per 100 g [60,61]. In the process of biochemical exploration and drug preparation, theobromine, an indispensable intermediate, serves as an important starting point for the creation of many drugs [62]. The pharmacological effects of theobromine include diuresis, myocardial stimulation, vascular relaxation, and smooth muscle relaxation, and it shows great potential in the treatment of related diseases [63,64]. In the food industry, theobromine contributes to the characteristic bitterness of chocolate and other cocoa-based products [4]. Additionally, as a phosphodiesterase inhibitor and weak adenosine receptor antagonist, theobromine has potential applications in the medical field [65,66,67].

Theophylline is a 1,3-dimethylxanthine, and its unique chemical structure stems from the methylation of the xanthine ring at the N-1 and N-3 positions, which endows it with wide-ranging application prospects in the biomedical field. Its primary effect is the relaxation of smooth muscles, particularly bronchial smooth muscles, via the inhibition of phosphodiesterase, which reduces the breakdown of cyclic adenosine monophosphate (cAMP), thereby promoting bronchial dilation [68,69]. Theophylline also enhances the excitability of the respiratory center, improving respiratory function and significantly increasing diaphragmatic contractility [70]. Theophylline can effectively improve heart function and blood circulation by enhancing myocardial contractility, increasing the heart rate, and promoting vasodilation [71]. Additionally, theophylline influences metabolic regulation by promoting the release of endogenous adrenaline and noradrenaline [72,73], thereby affecting lipid metabolism and energy production [74,75,76]. Theophylline also possesses anti-inflammatory properties [77], promotes airway ciliary movement, and has immunomodulatory effects on lymphocytes [78].

Paraxanthine (PX) is a high-value derivative of methylxanthine, distinguished by its unique chemical structure, with methyl groups attached at both the N-7 and N-1 positions of the xanthine ring. This distinct configuration contributes to its diverse biological activities. As a metabolite of caffeine, PX plays a neuroprotective role by stimulating ryanodine receptor channels, thereby preventing dopaminergic cell death [79,80,81]. In terms of applications, the characteristic bitterness and neurostimulatory properties of PX have led to its widespread use in the food and beverage industry, particularly in products such as tea, coffee, chocolate, and energy drinks [82,83].

Caffeine, a naturally occurring trimethylxanthine, is found in coffee, tea, and chocolate. Its chemical structure consists of a xanthine molecule methylated at the N-1, N-3, and N-7 positions [18,84]. Caffeine has a broad and notable range of physiological effects [85]. It competitively binds to adenosine receptors, inhibiting the effects of adenosine, which enhances neural excitability; improves alertness, attention, and reaction times; and imparts a sense of wakefulness and vitality [86]. Caffeine can stimulate fat decomposition, increase the concentration of free fatty acids in the blood, and increase the level of energy consumption, greatly helping people achieve healthy lifestyle goals. In addition, caffeine can enhance myocardial contractility, accelerate heart rate, and promote vasodilation, thus helping to improve heart function and blood circulation. These characteristics endow caffeine with important value in the control and management of hypertension, coronary artery disease, and other cardiovascular diseases [87,88].

## 3. Biosynthetic Pathways of Methylxanthines

At present, the synthetic pathways for methylxanthines include de novo synthesis and degradation-based synthesis using caffeine, theophylline, and theobromine as substrates [89,90]. Among these, the microbial N-demethylation of caffeine to synthesize various methylxanthines has garnered significant attention.

### 3.1. De Novo Biosynthesis of Methylxanthines

**Figure 2:**
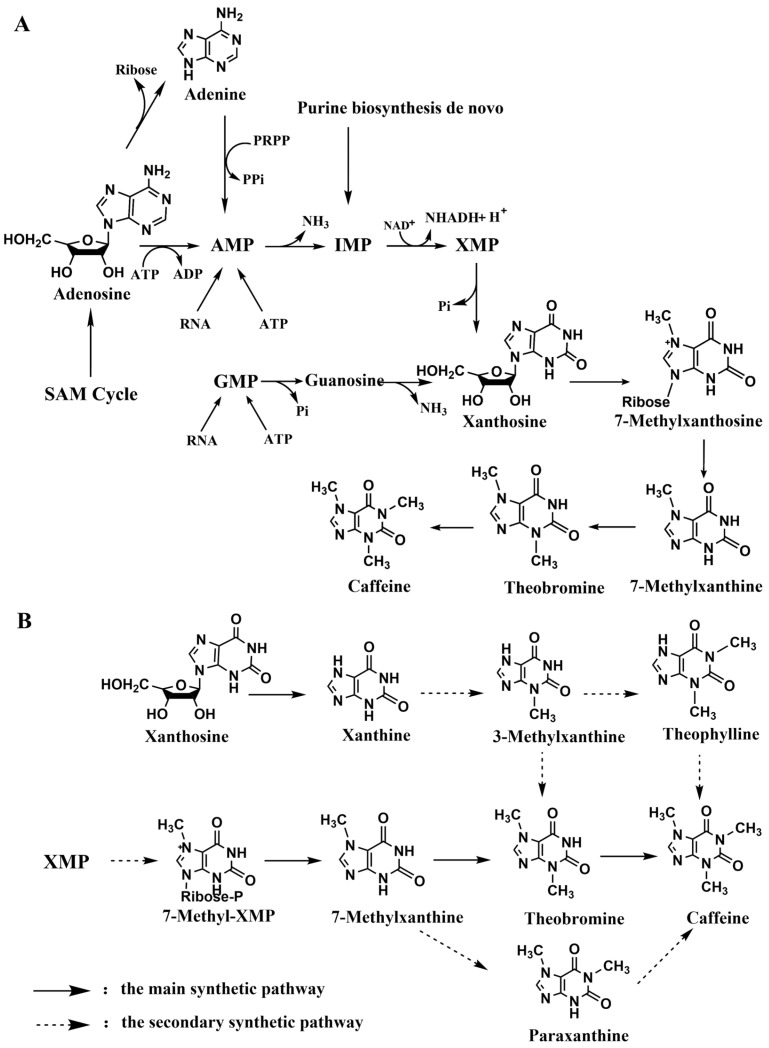
De novo biosynthetic pathway for caffeine. (**A**) The main biosynthetic pathway for caffeine in caffeine-producing plants. (**B**) The secondary (supplementary) biosynthetic pathway of caffeine in plants. SAM: S-adenosylmethionine, a key methyl donor in the biosynthesis of various compounds. AMP: Adenosine monophosphate, an intermediate in purine metabolism. IMP: Inosine monophosphate, a precursor in the purine biosynthesis pathway. XMP: Xanthosine monophosphate, an intermediate in the biosynthesis of xanthine derivatives. GMP: Guanosine monophosphate, involved in the synthesis of purine nucleotides.

In the biosynthesis of methylxanthines, xanthine serves as a critical precursor, undergoing methylation to produce various methylxanthine compounds. In plants, xanthine synthesis predominantly follows the de novo pathway, with key branches involving the metabolism of AMP and GMP (Figure 2A: This pathway involves the conversion of AMP and IMP to XMP and GMP, followed by key intermediates such as xanthosine, 7-methylxanthosine, and theobromine, ultimately producing caffeine. The SAM cycle provides the methyl group for methylation reactions at various steps. B: This pathway includes alternative routes involving intermediates such as xanthine, 3-methylxanthine, and theophylline, which contribute to caffeine formation.). These pathways are the primary sources of xanthine in tea plants. S-adenosylmethionine (SAM) plays a pivotal role in methylation reactions, participating in the production of methylxanthines such as caffeine [91].

The caffeine synthesis pathway in plants, which is based on the synthesis of xanthine adenosine and the subsequent formation of 7-methylxanthine ribonucleoside through the action of N-methyltransferase, has been elucidated. This compound is subsequently hydrolyzed by nucleoside hydrolases to generate 7-MX, which is further methylated by methyltransferases to form theobromine and caffeine [92,93]. Owing to the relatively low substrate specificity of methyltransferases, secondary pathways also exist in plants. As shown by the dashed lines in Figure 2B, however, these pathways in plants cannot be stopped after the production of high-value monomethylxanthines; they continue to add methyl groups to the high-value monomethylxanthines and eventually produce theobromine and caffeine, so monomethylxanthines can exist only at very low levels as intermediate products in plants. These compounds are present in commonly consumed beverages such as tea and coffee, being metabolized in the human body to perform various functions [22,45].

### 3.2. Synthesis of Methylxanthines Through Caffeine N-Demethylation

Caffeine is toxic to most bacteria and invertebrates; however, certain bacteria and fungi, including the species *Pseudomonas* sp., *Pseudomonas putida*, *Trichoderma* sp., *Fusarium oxysporum*, *Stemphyllium* sp., *Aspergillus tamarii*, and *Penicillium*, have evolved the ability to metabolize caffeine [94,95,96,97,98,99,100,101]. For example, among the seven strains of microorganisms isolated from Pu-erh tea, *Aspergillus sydowii* was proven to degrade caffeine and convert it into theophylline, while *Aspergillus ustus* and *A. tamarii* both showed the ability to degrade theophylline in liquid culture.

In humans, caffeine, theobromine, and other methylxanthines are metabolized primarily through N-demethylation and oxidation by cytochrome P450 enzymes in the liver. Similarly, in microorganisms, there are two parallel pathways for the metabolism of caffeine: the theobromine pathway and the theophylline pathway. These processes involve several N-demethylases and oxidases [102,103,104]. Enzymes encoded by genes isolated from *Pseudomonas putida* CBB5 (*ndmA*, *ndmB*, *ndmC*, *ndmD*, and *ndmE*) are responsible for the entire demethylation pathway [45,105,106,107]. In addition, the *cdhABC* genes, found in the *Pseudomonas* strain CBB1, are associated with the oxidation of caffeine.

The biosynthetic pathway for methylxanthine derivatives is catalyzed mainly by NdmABDE, where NdmA and NdmB are specific N-demethylases that act at the N-1 and N-3 positions, respectively. NdmC is responsible for the specific removal of the methyl group at the N-7 position. NdmD, an NADH-dependent reductase, transfers electrons, whereas NdmE plays a supporting role, enhancing the catalytic efficiency of these enzymes [108,109].

**Figure 3:**
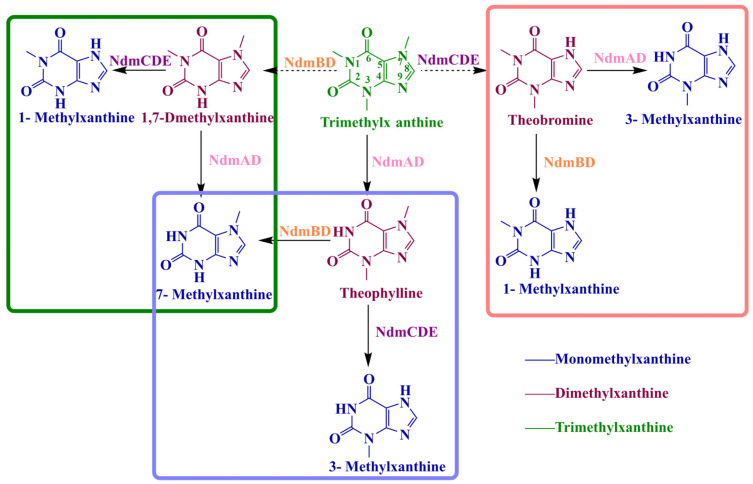
Structures of methylxanthine derivatives and their biosynthetic pathways. The blue structures represent monomethylxanthines, the purple structures represent dimethylxanthines, and the green structures represent trimethylxanthines. The figure illustrates the degradation of caffeine to various methylxanthine derivatives through a series of N-demethylation reactions catalyzed by the Ndm enzymes. In this process, NdmB and NdmD specifically catalyze N3-demethylation of methylxanthines, NdmA and NdmD catalyze N1-demethylation, and NdmC requires the formation of the NdmCDE complex with NdmD and NdmE to specifically catalyze N7-demethylation.

To date, a significant number of research studies and applications have focused on improving the specificity of N-demethylase active sites and enhancing the catalytic efficiency of these enzymes in the biosynthesis of high-value methylxanthine derivatives. In the biological degradation of caffeine to produce high-value methylated xanthine derivatives, caffeine is N-demethylated by various N-demethylases to generate monomethylxanthines and dimethylxanthines (Figure 3). Among these, relatively high production yields suitable for industrial-scale production have been achieved for 7-MX and 3-MX, making this a promising approach for the biosynthesis of high-value methylxanthines.

## 4. Advancements in Methylxanthine Biosynthesis Optimization

Researchers have made some significant progress in optimizing the biosynthesis of methylxanthine, especially in terms of increasing production. The following is a brief introduction to the research progress in this field (Table 1).

### 4.1. Advancements in the Synthesis of 1-Methylxanthine

To date, the biosynthesis of 1-methylxanthine has been studied mainly in *Escherichia coli*, in which the activity of 3-N-demethylase has been increased mainly by the mutation of *ndmA*. Mills et al. generated two mutants, nmdA3 and nmdA4, through targeted mutation of *ndmA*, and *ndmA3* presented increased 3-N-demethylase activity [110,111,112]. On this basis, Ryan M. Summers constructed an *E. coli* strain capable of producing 1-MX, utilizing a strain with *ndmA3* and incorporating *ndmD* reductase. This strain was able to synthesize 1-MX from theobromine. Additionally, by overexpressing the formaldehyde degradation gene *frmAB* from *E. coli*, a cofactor recycling system was established, eliminating formaldehyde, a byproduct produced during the synthesis of 1-MX. The system was capable of completely converting 10 mM theobromine in 5 h, achieving a conversion efficiency of 97.9% in a 7 L fermenter, with a final yield of 1.5 g/L 1-methylxanthine [32].

### 4.2. Advances in the Synthesis of 3-Methylxanthine

In a study from Xiaohui Liu, seven microbial strains were isolated from Pu-erh tea. Initially, five strains capable of utilizing theobromine were obtained from a selective medium containing high concentrations of theobromine. Afterward, the function of the catabolism of theobromine was tested in a special medium, and four strains that could decompose theobromine were obtained. The decomposition efficiency of *A. sydowii* PT-2 and *A. tamarii* PT-7 was greater than that of the other strains, with the decomposition efficiency of *A. sydowii* PT-2 reaching 64.5% and that of *A. tamarii* PT-7 reaching 94.3%. Metabolite analysis confirmed that *A. sydowii* could N-demethylate caffeine at the N-7 position to produce theobromine [45].

Algharrawi et al. utilized the *ndmA* and *ndmD* genes from *Pseudomonas putida* to engineer an *E. coli* strain capable of producing 3-MX from theobromine [22,113]. In a study by Mani Subramanian et al., increasing the copy number of the *ndmA* and *ndmD* genes in *E. coli* contributed to the high-yield production of 3-MX from theobromine [113].

In a study by Chang Liu et al., high yields of 7-MX were achieved during the development of their production process, prompting them to also explore the synthesis of 3-MX. Owing to the very low solubility of theobromine, which makes it unsuitable for production via large-scale fermentation, they chose to use theophylline as the substrate for 3-MX production. By utilizing the ndmA and ndmD enzymes, the engineered strain was able to convert theophylline to 3-MX, achieving a final yield of 22.96 ± 0.81 mM. This represents the highest known biosynthetic yield of 3-MX to date [32].

### 4.3. Advances in the Synthesis of 7-Methylxanthine

In the study by Ryan M. Summers, the process for the production of 7-MX from caffeine was first explored by identifying the N-demethylase gene cluster, consisting of NdmA, NdmB, NdmC, NdmD, and NdmE, sourced primarily from *Pseudomonas putida* and capable of metabolizing caffeine to xanthine. In vitro enzyme characterization revealed that NdmA is responsible for the N-1-demethylation of caffeine to theobromine; NdmB catalyzes the N3-demethylation of theobromine to 7-MX, and the NdmCDE complex facilitates the N7-demethylation of 7-MX to xanthine [23,105,114,115].

Within the NdmCDE complex, NdmC was clearly identified as the enzyme responsible for N7 demethylation, whereas NdmE plays a structural support role and does not possess catalytic activity. NdmD is a highly specific reductase for Ndm enzymes and plays a crucial role in the biocatalysis of N-demethylation by transferring electrons to NdmA, NdmB, and NdmC. This process is essential for efficient N-demethylation.

By obtaining the *ndmA* mutant *ndmA4*, which can N3-demethylate caffeine to produce PX while retaining activity for N1-demethylation of PX, researchers further optimized the system. However, NdmA4 has a low catalytic efficiency, so it was not used in subsequent experiments. Instead, a mixed microbial culture system was used, in which strains overexpressing the NdmA and ndmDP1 genes were first used to degrade caffeine to produce theobromine, and then strains overexpressing the NdmB and ndmDP1 genes were used to degrade theobromine to produce 7-methylxanthine. This system successfully converted caffeine to 7-MX using theobromine as an intermediate. After the ratio of the two strains and their cell densities was optimized, the optimal ratio of 50A:50B and an OD_600_ of 50 were established. In the large-scale conversion system, 2.5 mM caffeine was completely converted to 2.14 mM 7-MX and 0.235 mM theobromine, achieving 85.6% conversion to 7-MX and 9.4% conversion to theobromine. A recovery efficiency of 93.30% was achieved, resulting in 153.3 mg of 7-MX powder [112].

Mani Subramanian et al., on the basis of a previously established versatile biosynthetic process capable of converting theobromine to 3-MX and caffeine to theobromine [23,116], further explored the use of theobromine as a substrate for 7-MX production. They first examined the impact of varying the copy numbers of the *ndmB* and *ndmD* genes in *E. coli* on the efficiency of the conversion of theobromine to 7-MX. When the ratio of *ndmB* to *ndmD* was 50:50, a 100% conversion of theobromine to 7-MX was achieved, which was 10% more efficient than the conversion achieved with a 40:60 *ndmB* to *ndmD* ratio.

They also optimized the initial cell density and discovered that when the cell concentration was 15 mg/mL, the 100% conversion of theobromine to 7-MX was achieved within 60 min. As the initial cell concentration decreased, the time required for complete conversion of theobromine increased. Ultimately, they successfully recovered high-purity 7-MX powder, achieving a yield of 0.72 mg/mg [117].

In a recent study by Chang Liu et al., caffeine from coffee grounds was utilized as an ideal substrate, reducing production costs by recycling coffee waste. Researchers constructed a biosynthetic pathway for 7-MX production by incorporating *ndmA*, *ndmB*, and a modified *ndmD* gene, which are involved in the conversion of caffeine to 7-MX. To overcome bottlenecks, they employed a cofactor regeneration system composed of *frmA*, *frmB*, and *FDH* to regenerate NADH.

This study explored the effects of various fermentation factors, including dissolved oxygen levels, surfactant addition, initial cell OD value, and initial substrate concentration on the *E. coli* fermentation process for 7-MX production. After these factors were optimized, a 7-MX yield of 8.37 g/L was achieved with a product purity of 98.9%, which is the highest known yield of 7-MX to date [32].

### 4.4. Advances in the Synthesis of Paraxanthine

PX is a purine alkaloid derivative of caffeine that is produced through the action of an N-3-demethylase. It has significant medicinal value but its chemical synthesis often suffers from poor demethylation specificity, leading to low product purity. However, in the biosynthetic process, the selection of specific enzymes can increase the microbial production and purity of PX. The native N-demethylase NdmA exhibits weak N3-demethylation activity, converting approximately 1~2% of caffeine to PX and 13% of theobromine to 1-MX [113,116].

In the study by Ryan M. Summers, the copy numbers of *ndmA4* and *ndmD* were increased in *E. coli*, and amino acids 1–266 of *ndmD* were truncated to generate *ndmDP1*, an enzyme with increased N-demethylase activity. An NADPH regeneration system was also established [113,118]. This approach not only improved the enzymatic activity but also eliminated the negative effects of formaldehyde, a byproduct, on *E. coli* growth and the environment [119,120,121,122,123,124]. As a result, the engineered *E. coli* strain was able to produce 181 ± 5 μM PX within 5 h. Further optimization of the reaction conditions enabled the efficient conversion of caffeine to PX, with 300 mg of caffeine converted to 114.5 mg of PX. High-performance liquid chromatography (HPLC) was used to separate and purify the PX powder, yielding 104.1 mg with a purity of 90.9%, achieving the high-purity biosynthesis of PX [33].

In a recent study by Chang Liu et al., theobromine was used as a substrate to produce 1-MX, employing the *ndmB* and *ndmD* enzymes. The 1-MX product yield was 2.27 ± 0.03 mM; the yield was limited due to the weak N-1-demethylation activity of *ndmB* on methylxanthines. To increase the efficiency of the N-demethylation process, they introduced a NdmA mutant, NdmA4, but the yield of PX was still low at only 0.13 mM. Therefore, improving the substrate recognition capability of NdmB or further modifying NdmB may be a promising approach to increase its capacity for producing PX [32].

**Table 1:** Strategies and methods for the production of methylxanthines via microbial fermentation.

Modification Method	Strain	Strategy	Substrate	Product	Yield (g/L)	Reference
Enhanced biosynthetic pathway with reutilization of byproducts to increase yield	*E. coli* BL21(DE3)	Targeted mutation to obtain the high-efficiency enzyme *ndmA3*, combined with *ndmD*, and introduction of *frmAB* for the cofactor recycling system	Theophylline	1-MX	1.5	[33]
Enhanced biosynthetic pathway	*E. coli* BL21(DE3)	Introduction of the enzymes *ndmB* and *ndmD* into the cell, with the cofactor recycling system	Theophylline	1-MX	0.34	[110]
Enhanced biosynthetic pathway	*E. coli* BW25113	Use of efficient *ndmA* and *ndmD* enzymes	Theophylline	3-MX	3.8	[32]
Enhanced biosynthetic pathway	*E. coli* BL21(DE3)	Use of different copy numbers of *ndmA* and *ndmD*, increasing the *ndmD* copy number to enhance intracellular soluble *ndmD* levels	Theophylline	3-MX	0.14	[113]
Enhanced biosynthetic pathway with reutilization of byproducts to increase yield	*E. coli* BL21(DE3)	Targeted mutation to obtain high-efficiency enzyme *ndmA4*, and use of a mixed culture system with theobromine as an intermediate	Caffeine, Theobromine	7-MX	0.15	[112]
Enhanced biosynthetic pathway with optimized microbial ratio	*E. coli* BL21(DE3)	Optimized initial cell density and copy numbers of the *ndmB* and *ndmD* enzymes	Theobromine	7-MX	0.13	[117]
Enhanced biosynthetic pathway with cofactor recycling system to regenerate NADH and eliminate bottlenecks	*E. coli* BW25113	Introduction of *ndmA*, *ndmB*, and modified *ndmD* genes, along with *frmA*, *frmB*, and FDH for cofactor regeneration system	Caffeine	7-MX	8.37	[32]
Enhanced biosynthetic pathway with optimized enzyme copy numbers and NADPH regeneration system	*E. coli* BL21(DE3)	Increased the copy numbers of *ndmA4* and *ndmD*; truncate the first 266 amino acids of *ndmD* to generate high-efficiency enzyme *ndmDP1*; establish NADPH regeneration system	Caffeine	PX	0.12	[34]
Enhanced biosynthetic pathway with cofactor recycling system and high-efficiency *ndmA4* mutant	*E. coli* BW25113	Introduction of the *ndmB* and *ndmD* enzymes and cofactor recycling system, and use of the *ndmA4* mutant for high-efficiency enzyme production	Theophylline	PX	0.02	[32]

## 5. Conclusions and Future Perspectives

Methylxanthine derivatives, a class of compounds with significant biological activities, have demonstrated broad potential for application and promising prospects in various fields, including medicine, healthcare, and food. The exploration of their applications continues to deepen, with these derivatives being utilized in the pharmaceutical industry for the development of novel drugs and adjunctive therapies to address pressing challenges such as cardiovascular diseases, diabetes, and neurodegenerative disorders. In the cosmetics industry, their antioxidant properties have led to the creation of innovative antiaging and skin-whitening products.

Current research focuses on enhancing the production efficiency and purity of methylxanthine derivatives through approaches such as the genetic engineering of microorganisms, optimization of enzyme reaction conditions, and the use of immobilized enzyme technology [125]. Efforts to achieve industrial-scale production are centered on optimizing production processes, extending the industrial supply chain, and fostering international collaboration and market promotion. These initiatives aim to reduce production costs, increase yields, enhance industrial competitiveness, and improve product visibility and market share.

In the future, with the continuous advancement of technology and the ongoing expansion of global markets, research on and the application of methylxanthine derivatives will further evolve, driving innovation and transformation in related industries. Additionally, strengthening international cooperation and exchanges will inject new vitality and momentum into the development of this field.

## Data Availability

Not applicable.

## Abbreviations

The following abbreviations are used in this manuscript:

1-MX	1-methylxanthine
3-MX	3-methylxanthine
7-MX	7-methylxanthine
PX	Paraxanthine
*E. coli*	*Escherichia coli*
